# Sensing succinate: SUCNR1 as a context-dependent metabolic and cellular signal integrator

**DOI:** 10.3389/fmolb.2026.1815835

**Published:** 2026-04-01

**Authors:** Aenne-Dorothea Liebing, Claudia Stäubert

**Affiliations:** Rudolf Schönheimer Institute of Biochemistry, Medical Faculty, Leipzig University, Leipzig, Germany

**Keywords:** cancer, G protein-coupled receptor (GPCR), metabolism, pro- and anti-inflammatory immune responses, signal selectivity, signal transduction, succinate, succinate receptor 1 (SUCNR1)

## Abstract

Succinate (SUC), a central intermediate in the mitochondrial tricarboxylic acid (TCA) cycle, functions not only as a metabolic substrate but also acts as the endogenous ligand for succinate receptor 1 (SUCNR1), a G_i_- and G_q_ protein-coupled receptor. SUC accumulates when energy demand exceeds oxygen supply or during metabolic rewiring, including hypoxia, endurance exercise, inflammation, and tumor progression. SUC can be released into the extracellular space, reaching levels sufficient to activate SUCNR1. SUCNR1 is expressed in various tissues, including the kidney, liver, and adipose tissue, as well as in immune cells and cancer subtypes. Rather than functioning as a simple pro- or anti-inflammatory receptor, SUCNR1 acts as a metabolic signal integrator whose output is determined by G protein preferences, receptor trafficking, and the balance between intra- and extracellular SUC pools. In immune cells, particularly macrophages, SUCNR1 signaling promotes either inflammatory activation or resolution depending on the metabolic state. In metabolic tissues and cancer, SUCNR1 coordinates adaptive responses to nutrient and oxygen stress while shaping the tissue microenvironment. Here, we review recent advances in SUC-SUCNR1 signaling across immune and metabolic systems, discuss unresolved controversies regarding signaling selectivity and spatial encoding, and evaluate the therapeutic opportunities and challenges of targeting this metabolic checkpoint.

## Introduction

Succinate (SUC) is a central intermediate of cellular metabolism, as part of the mitochondrial tricarboxylic acid (TCA) cycle, being directly linked to the electron transport chain via succinate dehydrogenase (SDH) (Murphy and Chouchani, 2022; Fernández-Veledo et al., 2021; Pålsson-McDermott and O'Neill, 2025; Huang et al., 2024). SUC is also produced via the γ-aminobutyric acid shunt and via succinyl-CoA linked to the metabolism of ketone bodies, branched-chain amino acids, odd-chain fatty acids, and heme (Tretter et al., 2016; Zhang and Lang, 2023; Ye et al., 2020; Fernández-Veledo et al., 2024). During inflammation, the macrophage-derived SDH inhibitor itaconate accumulates and directly impacts SUC levels (Atallah et al., 2025; Lampropoulou et al., 2016).

Metabolic disturbances that uncouple energy demand from oxygen availability increase intra- and extracellular SUC levels and alter pH, influencing SUC transport and signaling (Murphy and O'Neill, 2018; Chouchani et al., 2014; Mills and O'Neill, 2014; Tannahill et al., 2013). Such conditions include hypoxia, inflammation, physical exercise, or cancer. Although normally confined to the mitochondrial matrix, SUC is exported under metabolic stress. SUC concentrations fluctuate widely, ranging from ∼2 to 30 µM in plasma and from ∼0.5 mM under normoxia to ∼6 mM under hypoxia within mitochondria (Grimolizzi and Arranz, 2018; Ariza et al., 2012). Gut microbial SUC production further adds to the system’s complexity (Fernández-Veledo and Vendrell, 2019).

Beyond its metabolic role, SUC itself acts as a signaling molecule via succinate receptor 1 (SUCNR1, GPR91), a G_i_- and G_q_-coupled receptor deorphanized in 2004 (He et al., 2004). EC_50_ values are in the low micromolar range, overlapping with plasma concentrations induced by metabolic stress. However, whether local tissue concentrations consistently reach levels sufficient for sustained receptor activation remains unclear. Because mitochondrial, cytosolic, and extracellular SUC pools are constrained by transport and diffusion barriers, defining SUC gradients across cellular compartments and tissues is critical to determine when SUCNR1 is physiologically engaged.

SUCNR1 is expressed in liver (He et al., 2004; Wittenberger et al., 2001; Littlewood-Evans et al., 2016; Mills et al., 2021), kidney (He et al., 2004; Wittenberger et al., 2001; Robben et al., 2009), adipose tissue (Regard et al., 2008; McCreath et al., 2015; Villanueva-Carmona et al., 2023), and innate immune cells (Rubic et al., 2008). In hepatocytes, it regulates glucose and lipid metabolism, whereas in Kupffer and stellate cells, it promotes inflammation and fibrosis (Nguyen et al., 2022; Cho, 2018; Li Y. H. et al., 2015; Marsal-Beltran et al., 2023; Yang et al., 2025). In the kidney, it increases renin release, thereby regulating blood pressure (Toma et al., 2008; Peti-Peterdi et al., 2013; Peti-Peterdi, 2010; Vargas et al., 2009). In white adipose tissue, SUCNR1 inhibits lipolysis and browning (McCreath et al., 2015; Villanueva-Carmona et al., 2023).

Synthetic ligands for SUCNR1 include *cis*-epoxysuccinate (CES) (Geubelle et al., 2017), compound **31** (Rexen Ulven et al., 2018), and several antagonists with variable species-specificity and potency (Haffke et al., 2019; Thomas et al., 2024; Guo et al., 2022; Bhuniya et al., 2011; Velcicky et al., 2020). While the SUCNR1-mediated signaling profiles of CES and SUC are comparable, compound **31** is a partial agonist that primarily activates G_i_ signaling (Rexen Ulven et al., 2018; Liebing et al., 2025). To date, ten cryo-EM structures of SUCNR1 in inactive (Haffke et al., 2019) and active (Wang et al., 2024; Liu et al., 2024; Li et al., 2024) conformations provide insights into ligand binding and receptor conformation, although the determinants governing selective G_i_ versus G_q_ coupling remain unclear. Resolving whether coupling preference depends on ligand concentration, receptor density, membrane environment, or trafficking will require structures of SUCNR1 bound to distinct G proteins under defined metabolic conditions.

Here, we highlight controversies in SUCNR1 signaling and summarize how spatially encoded SUC-sensing shapes immune and metabolic responses.

### SUCNR1 signaling dependence on cellular metabolism and subcellular location

**FIGURE 1:**
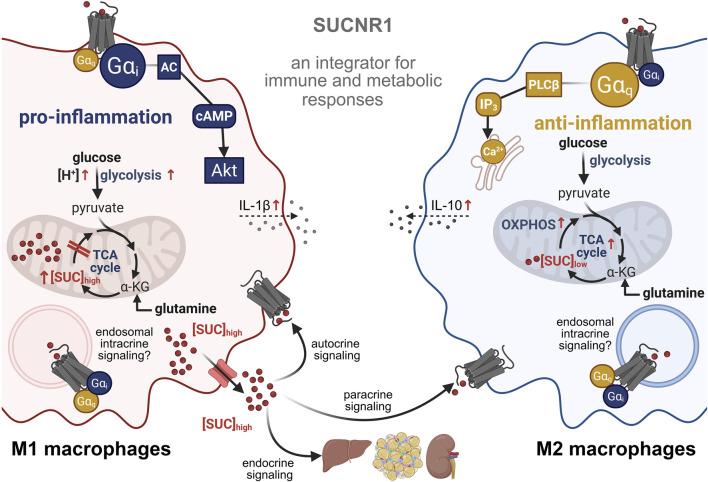
Succinate receptor 1 (SUCNR1) as a metabolic signal integrator in immune cells. Pro-inflammatory M1 macrophages rely on aerobic glycolysis and are major producers of succinate (SUC). Anti-inflammatory M2 macrophages favor oxidative phosphorylation (OXPHOS) and express higher levels of SUCNR1. SUCNR1 is a G_i_ and G_q_ protein-coupled receptor that senses SUC, which can accumulate both extracellularly and intracellularly. In addition to endocrine, paracrine, and autocrine signaling, SUCNR1 may also act as an intracrine SUC sensor that recognizes locally restricted SUC pools, although direct evidence is limited. Created with Biorender.

Intracellular SUC production depends on substrate availability, oxygen levels, and metabolic enzyme expression. For instance, glutamine causes increased oxygen consumption and TCA cycle flux relative to glucose (Liebing et al., 2025; Rabe et al., 2022). These parameters shape intra- and extracellular SUC levels, thereby modulating SUCNR1 signaling, positioning the receptor as a regulator of cellular energy homeostasis that limits excessive respiration under high-energy states (Figure 1).

In accordance with this metabolic sensitivity, SUCNR1-mediated G_i_ signaling is robustly observed, whereas evidence for G_q_ coupling remains variable across experimental systems (He et al., 2004; Robben et al., 2009; Liebing et al., 2025; Rabe et al., 2022; Gilissen et al., 2016; Gilissen et al., 2015; Sabadell-Basallote et al., 2024; Trauelsen et al., 2021; Sundström et al., 2013; Abdelmoez et al., 2023). Several aspects are relevant in this context. Glutamine, present in most cell culture media, increases TCA cycle flux and basal SUC levels, shifting SUCNR1 signaling towards reduced G_q_ activation and Ca^2+^ release, while enhancing receptor internalization (Liebing et al., 2025). This metabolic background likely contributes to the inconsistent detection of SUCNR1-dependent G_q_ signaling across experimental systems.

Activation of phospholipase Cβ (PLCβ) downstream of Gα_q_ generates inositol 1,4,5-trisphosphate (IP_3_) and diacylglycerol. Gα_i_-derived Gβγ subunits modulate Ca^2+^ release and IP_3_ formation, but robust PLCβ activation requires active Gα_q_, indicating that effective Ca^2+^ responses depend on coordinated G_i_ and G_q_ engagement (Liebing et al., 2025; Sundström et al., 2013; Pfeil et al., 2020; Kankanamge et al., 2021; Ubeysinghe et al., 2023). Notably, SUCNR1-mediated G_q_ signaling has also been observed without measurable Ca^2+^ release, indicating alternative downstream effectors (Liebing et al., 2025).

SUCNR1 exhibits rapid internalization and turnover, with limited arrestin recruitment (Geubelle et al., 2017; Gilissen et al., 2015). Activation engages multiple kinase pathways, including extracellular signal-regulated kinase 1/2 (ERK1/2), protein kinase B (PKB/Akt), protein kinase C (PKC), AMP-activated protein kinase (AMPK), and c-Jun N-terminal kinase (JNK), linking SUC-sensing from plasma membrane-localized, internalized, or pre-existing intracellular receptor pools to varied cellular outcomes, such as the promotion of mitochondrial fission via a PKC/ERK-dependent pathway (Villanueva-Carmona et al., 2023; Liebing et al., 2025; Rabe et al., 2022; Trauelsen et al., 2021; Zhang et al., 2025; Keiran et al., 2019; Lu et al., 2018).

SUCNR1 signaling is highly dynamic, shaped by G protein-coupling preference, cellular metabolic state, and its subcellular localization: G_i_ protein activation at the plasma membrane persists in endosomes, whereas miniG_q_, but not miniG_i_, is recruited to late endosomes (Liebing et al., 2025). MiniG proteins are engineered, truncated G protein GTPase domains originally developed to stabilize GPCRs in their active conformation for structural studies and are now widely used as conformational probes to report receptor activation in living cells (Wan et al., 2018; Nehmé et al., 2017; Carpenter and Tate, 2017).

As endosomal phosphoinoside composition differs from that of the plasma membrane, PLCβ-mediated IP_3_ formation is hampered (Posor et al., 2022; Daly and Plouffe, 2025). Gα_q_ signaling from endosomes has been reported, although the underlying molecular mechanisms remain unclear (Daly and Plouffe, 2025). Because SUCNR1 exhibits G_q_-dependent signaling in the absence of detectable IP_3_ and Ca^2+^ responses, this signal may originate from endosomal compartments rather than the plasma membrane (Liebing et al., 2025). Gα_q_ also localizes to lysosomes and autophagic compartments, where it regulates assembly and activation of mammalian target of rapamycin complex 1 (mTORC1), suppressing autophagy when nutrients are abundant (Cabezudo et al., 2021). Whether SUCNR1 engages this axis remains an open and intriguing question. In addition to the plasma-membrane and endosomal location of Gα_q_, there is evidence for the presence of Gα_q_ at mitochondrial membranes, where it increases respiratory capacity, ATP production, and oxidative-phosphorylation (OXPHOS)- dependent growth (Benincá et al., 2014; Chakraborty et al., 2023).

Both Gα_q_ and Gα_i_ are present in intracellular membranes, supporting compartmentalized GPCR signaling (Bastin et al., 2015; Garcia-Marcos et al., 2011; Lyssand et al., 2007; Jang et al., 2024; Plouffe et al., 2020; Fasciani et al., 2022; Bock et al., 2025). Thus, spatially encoded SUCNR1 signaling may fine-tune receptor responses to metabolic state and intra-versus extracellular SUC levels. Beyond established endocrine, paracrine, and autocrine roles, SUCNR1 may function as an intracrine SUC sensor responsive to spatially restricted SUC pools, analogous to the mechanisms described for the free fatty acid receptor 4, although direct evidence remains limited (Fernández-Veledo et al., 2021; Marsal-Beltran et al., 2023; Abdelmoez et al., 2023; O'Brien et al., 2026) (Figure 1).

SUC dynamics are highly compartmentalized, with intracellular and extracellular levels fluctuating independently or in concert, creating distinct states that shape SUCNR1 activation and downstream responses (Guo et al., 2020).

### Metabolic state control of SUCNR1-mediated immunomodulation

Inflammatory conditions are consistently associated with elevated SUC levels both systemically and locally within affected tissues, including multiple inflammatory and metabolic diseases, such as Crohn’s disease, ulcerative colitis, diabetes, obesity, hypertension, atherosclerosis, cardiac hypertrophy, hepatic fibrosis, rheumatoid arthritis, pulmonary fibrosis, periodontitis, osteoporosis, or endometriosis (Fernández-Veledo et al., 2024; He et al., 2004; Rubic et al., 2008; Li Y. H. et al., 2015; Guo et al., 2022; Bauset et al., 2022; Macias-Ceja et al., 2019; Ceperuelo-Mallafré et al., 2019; Sapieha et al., 2008; van Diepen et al., 2017; Serena et al., 2018; Yuan et al., 2024; Osuna-Prieto et al., 2021; Aguiar et al., 2010; Aguiar et al., 2014; Saraiva et al., 2018; He et al., 2024; Wu et al., 2025; Tian et al., 2024). In many of these settings, SUCNR1 amplifies inflammatory responses (Jia and Wang, 2025), although protective roles have also been reported under certain metabolic conditions. SUCNR1 is enriched in myeloid cells, particularly macrophages (Keiran et al., 2019; van Diepen et al., 2017; Trauelsen et al., 2017) and dendritic cells (Rubic et al., 2008; Saraiva et al., 2018), where it governs immune cell polarization, chemotaxis, and metabolic programming (Littlewood-Evans et al., 2016; van Diepen et al., 2017). SUC itself is a central immunometabolite that shapes macrophage metabolic adaptation during inflammation and can exert either pro- or anti-inflammatory effects depending on the prevailing metabolic environment (reviewed in Atallah et al., 2025).

Macrophage subsets exhibit distinct metabolic and signaling profiles. Pro-inflammatory M1 macrophages primarily rely on aerobic glycolysis and are the dominant producers of SUC, whereas anti-inflammatory M2 macrophages favor OXPHOS and express higher levels of SUCNR1 (Tian et al., 2024; Martinez et al., 2008; Natoli et al., 2021; Viola et al., 2019; Liu et al., 2021; Dai et al., 2025) (Figure 1). This discrepancy highlights the need to consider metabolic environment and signaling selectivity when interpreting SUC-SUCNR1 biology. Intracellular SUC reinforces M1 polarization by stabilizing hypoxia-inducible factor 1α (HIF-1α) while also amplifying inflammatory responses by increasing SUCNR1 expression and SUCNR1-dependent interleukin (IL) 1β secretion, linking receptor signaling to metabolic state and immune cell function through a positive feedback loop (Pålsson-McDermott and O'Neill, 2025; Tannahill et al., 2013; Littlewood-Evans et al., 2016; Keiran et al., 2019; Jia and Wang, 2025; Caslin et al., 2020; Lumeng et al., 2008) (Figure 1). In the chronically inflamed brain, inflammatory mononuclear phagocytes release SUC, which activates SUCNR1 on neural stem cells, causing upregulation of solute carrier family 13 (SLC13) transporters, thereby enhancing SUC uptake and linking extracellular sensing to intracellular metabolic remodeling (Peruzzotti-Jametti et al., 2018). In pro-inflammatory macrophages, SLC13A3-mediated Na^+^ uptake elevates cytosolic SUC and sustains inflammatory polarization (Fremder et al., 2021). Elevated serum and fecal SUC levels in patients with inflammatory bowel disease further support that increased extracellular SUC sustains SUCNR1-driven inflammatory signaling *in vivo* (Fernández-Veledo et al., 2025).

Additionally, emerging evidence highlights a protective role for the SUC-SUCNR1 axis in M2 macrophage polarization and inflammation (Trauelsen et al., 2021). Although M1 macrophages produce SUC, SUCNR1 is enriched on M2 macrophages, where it promotes anti-inflammatory programs (Figure 1). Metabolic balance is a key determinant of these outcomes, as a high α-ketoglutarate-to-SUC ratio favors resolution, whereas a low ratio promotes inflammation (Dai et al., 2025; Liu et al., 2017). During acute hypoxia, macrophages accumulate SUC due to a shift toward glycolysis and TCA cycle disruption (Zhang et al., 2023). During ischemia-reperfusion, macrophages transition toward oxidative metabolism and M2 phenotypes, associated with a decline of intracellular SUC relative to extracellular pools released by apoptotic cells (Dal-Secco et al., 2015).

SUC-treated macrophages attenuate colitis *in vivo* (Park et al., 2021). In mesenchymal stem cells, intracellular SUC accumulation promotes IL-10 and prostaglandin E2 (PGE_2_) release, driving M2 repolarization, a switch further reinforced through SUCNR1-dependent G_q_-PLCβ signaling (Trauelsen et al., 2021; Peruzzotti-Jametti et al., 2018; Yuan et al., 2022). Myeloid-specific Sucnr1 deletion shifts murine adipose macrophages toward a pro-inflammatory profile, whereas global knockout reduces macrophage infiltration (Keiran et al., 2019; van Diepen et al., 2017). Under physiological conditions, extracellular SUC may promote anti-inflammatory phenotypes in adipose tissue-resident macrophages via coordinated G_i_ and G_q_ signaling, preserving metabolic homeostasis and counteracting potential inflammatory signals (Trauelsen et al., 2021). In obesity and type 2 diabetes, sustained SUC accumulation and extracellular acidification may enhance SUC import, dampen SUCNR1-G_q_ signaling, and thereby shift SUCNR1 toward pro-inflammatory functions, inducing macrophage recruitment (van Diepen et al., 2017).

In macrophages, SUCNR1 engages both G_i_- and G_q_-mediated signaling pathways. G_i_ supports metabolic reprogramming associated with M1 polarization to meet the energetic demands of immune surveillance and tissue remodeling (Liebing et al., 2025; Dai et al., 2025; Li X. et al., 2015; Vural et al., 2019; Langston et al., 2017) (Figure 1). G_q_ activation modulates glycolytic flux and can suppress NLRP3 inflammasome activation in macrophages (Liebing et al., 2025; Dai et al., 2025; Kong et al., 2024). While ERK1/2 can exert divergent immune effects, Akt signaling is reduced in M2 macrophages and further dampened by SUCNR1-G_q_ signaling, potentially stabilizing M2 phenotypes, consistent with reported M2 hyperpolarization (Liebing et al., 2025; Trauelsen et al., 2021; Vergadi et al., 2017) (Figure 1).

Collectively, SUCNR1 does not dictate macrophage polarization in a binary manner but tunes responsiveness to prevailing metabolic cues. Acute metabolic stress and intracellular SUC accumulation favor G_i_-driven pro-inflammatory programs, whereas in metabolically balanced environments, G_q_ signaling stabilizes anti-inflammatory phenotypes. This metabolic integrator model reconciles divergent findings across tissues and disease settings.

### SUCNR1 beyond immune cell regulation

Beyond macrophage polarization, SUC-SUCNR1 signaling influences tissue remodeling programs.

In human skeletal muscle, SUC drives exercise-induced fast-to-slow fiber transitions via SUCNR1-expressing M2-like macrophages in a paracrine manner (Wu et al., 2025). Acute exercise increases SUC locally in mouse skeletal muscle and transiently elevates plasma SUC in humans, where SUCNR1 associates with M2-polarized macrophages, unlike its low and polarization-independent expression in mice (Abdelmoez et al., 2023).

In bone, stromal SUC signals via SUCNR1 on osteoclast lineage cells to drive osteoclastogenesis (Guo et al., 2017). Most evidence for this mechanism derives from inflammatory and metabolic disease models, including type 2 diabetes and rheumatoid arthritis, where elevated SUC levels correlate with increased osteoclast differentiation and bone resorption (Littlewood-Evans et al., 2016; Guo et al., 2017). In rheumatoid arthritis, SUC accumulates in inflamed joints and contributes to SUCNR1-dependent inflammatory signaling that further enhances osteoclast activity and joint damage (Littlewood-Evans et al., 2016; Saraiva et al., 2018).

In the liver, SUCNR1 mediates a metabolically tuned stress response. Global Sucnr1 knockout reduces Kupffer cell inflammation under basal conditions, whereas elevated extracellular SUC in uncoupling protein 1 (UCP1)-deficient mice drives SUCNR1-dependent inflammatory signaling in stellate cells and macrophages (Mills et al., 2021). Increasing brown and beige adipocyte content counteracts this effect, highlighting a UCP1-SUC-SUCNR1 axis that regulates hepatic immune cell infiltration in response to metabolic state (Mills et al., 2021). Consistent with this context dependence, SUCNR1 also supports an early anti-inflammatory liver stress response that restrains steatosis and glycogen depletion while coordinating immune-metabolic repair, although with pro-fibrotic consequences (Marsal-Beltran et al., 2023).

In the small intestine, SUC-SUCNR1 signaling engages tuft cell type 2 innate lymphoid cell (ILC2) circuits, promoting IL-25-dependent ILC2 expansion and IL-13 production, thereby supporting type 2 immunity and epithelial barrier integrity (Ogulur et al., 2025; Schneider et al., 2019; Schneider et al., 2018; Nadjsombati et al., 2018; Lei et al., 2018).

In adipose tissue, hypoxia and hyperglycemia promote SUC release from adipocytes, and circulating SUC levels are elevated in type 2 diabetes and obesity (van Diepen et al., 2017). In adipocytes, G_i_-mediated signaling suppresses lipolysis, whereas G_q/11_ signaling is required for maintaining glucose homeostasis and normal plasma free fatty acid levels (Kimura et al., 2022). Adipocyte-specific Sucnr1 deletion in mice promotes white adipose tissue browning, whereas a global knockout alters adipose mass and disrupts glucose homeostasis, impairing SUC-mediated suppression of lipolysis (McCreath et al., 2015; Villanueva-Carmona et al., 2023; Kimura et al., 2022). This underscores its function in regulating energy storage and expenditure. In obesity and insulin-resistance, sustained extracellular SUC accumulation may shift SUCNR1 signaling toward pro-inflammatory and metabolic stress pathways, contributing to macrophage recruitment and adipose tissue inflammation.

In pancreatic β cells, SUCNR1 is upregulated under hyperglycemia, where SUC enhances glucose-stimulated insulin secretion via G_q_-PKC signaling. β-cell-specific Sucnr1 deletion impairs insulin release and glucose tolerance during high-fat feeding, linking SUCNR1 to compensatory hyperinsulinemia in early metabolic disease (Sabadell-Basallote et al., 2024).

### SUCNR1 in cancer

In cancer cells, SUC accumulates due to mutations in TCA cycle enzymes, hypoxic microenvironments, or metabolic alterations that favor aerobic glycolysis over OXPHOS (Zhang and Lang, 2023; Martínez-et al., 2020; Sant'Anna-Silva et al., 2021; Wise and Thompson, 2010). Cancer cells often reprogram their metabolism to meet increased demands for energy and anabolic processes (Mao et al., 2024; Vasan et al., 2020). Although many cancer cells rely on aerobic glycolysis, also known as the Warburg effect, to generate ATP, an intact mitochondrial metabolism remains essential for biosynthesis and TCA cycle anaplerosis (Vasan et al., 2020). Elevated intracellular SUC can additionally drive succinylation of proteins, a post-translational modification that is increased in cancer and inflammatory contexts and often suppresses mitochondrial enzyme activity while promoting glycolytic metabolism (Jiang et al., 2025; Qi et al., 2019; Yu et al., 2025; Hou et al., 2024). Whether SUCNR1 itself undergoes succinylation has not yet been reported. SUCNR1 exerts both tumor cell-intrinsic and microenvironmental effects. In cancer cells, SUCNR1 restrains mitochondrial respiration and reactive oxygen species (ROS) production via G_i_-dependent suppression of TCA cycle flux, promoting cell survival, particularly in glutamine-replete conditions (Rabe et al., 2022). SUCNR1 loss sensitizes to chemotherapeutics (Rabe et al., 2022). Elevated SUC stabilizes HIF-1α, enhancing glycolysis, angiogenesis, and tumor growth. Tumor-derived SUC modulates the tumor microenvironment through SUCNR1-dependent immunosuppressive signaling, activating phosphoinositide 3-kinase (PI3K)/Akt and HIF-1α, to promote M2 macrophage polarization, regulatory T cell expansion, and therapy resistance (Wu et al., 2020; Chen et al., 2026; Kinsella et al., 2025; Najm et al., 2022). In contrast, intracellular delivery of SUC via tumor-derived microparticles reprograms tumor-associated macrophages by enhancing glycolysis and protein succinylation, independent of SUCNR1 activation, thereby promoting M1-like polarization (Lu et al., 2025).

Although mechanistic details remain unclear, current evidence suggests that SUCNR1 functions as a metabolic checkpoint receptor that protects cancer cells from excessive metabolic stress and oxidative damage, thereby contributing to tumor survival in nutrient-rich and hypoxic environments.

## Discussion

SUCNR1 is a critical player at the interface between cellular metabolism and receptor signaling, uniquely positioned to sense metabolic perturbations and translate them into adaptive cellular responses. Rather than acting solely as a pro- or anti-inflammatory receptor, accumulating evidence indicates that SUCNR1 functions as a metabolic integrator regulated by three main factors: (i) the magnitude and origin of extracellular versus intracellular SUC pools, (ii) receptor localization and trafficking, and (iii) the overall metabolic state of the responding cell.

This framework helps reconcile apparently divergent findings across different tissues. Distinguishing extracellular SUC-SUCNR1 signaling from intracellular SUC-driven metabolic and epigenetic modifications is essential, as they arise from similar metabolic perturbations but operate through distinct mechanisms. Blurring these layers may contribute to apparent inconsistencies in the literature.

A central unresolved question is whether SUCNR1 primarily senses extracellular SUC or also responds to spatially restricted intracellular SUC pools. While compartmentalized and endosomal signaling is supported, direct evidence for intracellular SUCNR1 activation remains limited. Importantly, current evidence largely supports prolonged signaling from internalized receptors after extracellular activation, rather than direct activation by intracellular SUC pools. Whether intracellular SUC engages SUCNR1 within endosomes or other organelles, or whether endosomal signaling merely reflects prolonged, plasma membrane-initiated activity, remains unresolved. Determining ligand accessibility, receptor orientation, and compartmental SUC gradients will be essential to substantiate an intracrine sensing model and guide therapeutic strategies.

From a translational perspective, SUCNR1 presents both opportunity and complexity, as its broad tissue distribution and state- and compartment-specific signaling complicate global inhibition strategies. Therapeutic targeting holds promise for the treatment of inflammatory diseases, metabolic disorders, and cancer. However, selective modulation remains challenging. In immune cells, global inhibition could impair beneficial anti-inflammatory or tissue-repair functions, whereas in tumors, SUCNR1 inhibition may increase oxidative stress and compromise tumor cell survival. These opposing effects highlight the importance of precision approaches to enable selective therapeutic intervention. Cell-type- and compartment-specific targeting, as well as ligands that preferentially engage G_i_- or G_q_-mediated signaling pathways, may enable selective therapeutic modulation of SUCNR1. Achieving this will require a deeper understanding of the molecular, structural, and trafficking determinants that govern SUCNR1 coupling to distinct G proteins. Progress will depend on integrating structural biology, quantitative metabolite profiling, conditional genetic models, and spatially resolved signaling analyses.

Ultimately, SUCNR1 can be viewed as a programmable metabolic checkpoint that reflects the balance between cellular energy demand and the availability of nutrients and oxygen. Acute intracellular SUC accumulation may favor G_i_-driven pro-inflammatory programs, whereas balanced SUC gradients may preferentially engage G_q_- or resolution-linked pathways. Testing this framework will require controlled manipulation of SUC gradients, receptor localization, and G protein availability. Therapeutically, targeting may extend beyond receptor modulation by altering SUC availability itself through metabolic rewiring, transporter targeting, or microenvironmental pH control, thereby indirectly shaping SUCNR1 signaling, without directly interfering with receptor function.

## Conclusion

Despite significant advances, the integrative logic governing SUCNR1 signaling remains incompletely defined. Resolving how receptor localization, G protein-coupling preferences, and SUC gradients converge to shape downstream responses will require coordinated approaches that combine high-resolution spatiotemporal signaling analyses, quantitative assessment of SUC fluxes, and conditional, cell-type-specific genetic models.

Together, current evidence positions SUCNR1 as a dynamic metabolic checkpoint that integrates SUC availability with immune cell fate and tissue remodeling across immune and metabolic niches. Conceptualizing SUCNR1 as a programmable metabolic integrator rather than a binary inflammatory switch may resolve current controversies and enable rational, precision-based targeting of this metabolite-receptor axis.
